# RNA-seq and metabolomic analyses of Akt1-mediated muscle growth reveals regulation of regenerative pathways and changes in the muscle secretome

**DOI:** 10.1186/s12864-017-3548-2

**Published:** 2017-02-16

**Authors:** Chia-Ling Wu, Yoshinori Satomi, Kenneth Walsh

**Affiliations:** 1 Molecular Cardiology, Whitaker Cardiovascular Institute, Boston University School of Medicine, 715 Albany Street, W-611, Boston, MA 02118 USA; 2 Integrated Technology Research Laboratories, Takeda Pharmaceutical Co. Ltd., 26-1, Muraoka-Higashi 2-chome, Fujisawa, Kanagawa 251-8555 Japan

**Keywords:** Akt1, Muscle growth, Skeletal muscle, RNA-seq, Metabolomics, Secreted protein, Pentose phosphate pathway, Cell cycle, Inflammation, Regeneration

## Abstract

**Background:**

Skeletal muscle is a major regulator of systemic metabolism as it serves as the major site for glucose disposal and the main reservoir for amino acids. With aging, cachexia, starvation, and myositis, there is a preferential loss of fast glycolytic muscle fibers. We previously reported a mouse model in which a constitutively-active Akt transgene is induced to express in a subset of muscle groups leading to the hypertrophy of type IIb myofibers with an accompanying increase in strength. This muscle growth protects mice in various cardio-metabolic disease models, but little is known about the underlying cellular and molecular mechanisms by which fast-twitch muscle impacts disease processes and regulates distant tissues. In the present study, poly (A) + tail mRNA-seq and non-targeted metabolomics were performed to characterize the transcriptome and metabolome of the hypertrophic gastrocnemius muscle from *Akt1*-transgenic mice.

**Results:**

Combined metabolomics and transcriptomic analyses revealed that Akt1-induced muscle growth mediated a metabolic shift involving reductions in glycolysis and oxidative phosphorylation, but enhanced pentose phosphate pathway activation and increased branch chain amino acid accumulation. Pathway analysis for the 4,027 differentially expressed genes in muscle identified enriched signaling pathways involving growth, cell cycle regulation, and inflammation. Consistent with a regenerative transcriptional signature, the transgenic muscle tissue was found to be comprised of fibers with centralized nuclei that are positive for embryonic myosin heavy chain. Immunohistochemical analysis also revealed the presence of inflammatory cells associated with the regenerating fibers. Signal peptide prediction analysis revealed 240 differentially expressed in muscle transcripts that potentially encode secreted proteins. A number of these secreted factors have signaling properties that are consistent with the myogenic, metabolic and cardiovascular-protective properties that have previously been associated with type IIb muscle growth.

**Conclusions:**

This study provides the first extensive transcriptomic sequencing/metabolomics analysis for a model of fast-twitch myofiber growth. These data reveal that enhanced Akt signaling promotes the activation of pathways that are important for the production of proteins and nucleic acids. Numerous transcripts potentially encoding muscle secreted proteins were identified, indicating the importance of fast-twitch muscle in inter-tissue communication.

**Electronic supplementary material:**

The online version of this article (doi:10.1186/s12864-017-3548-2) contains supplementary material, which is available to authorized users.

## Background

The ability of skeletal muscle to quickly adapt its structure and function to external stimuli allows for diverse movement, and this is associated with systemic metabolic and cardiovascular changes to meet the changing functional demands of the muscle. The mass and composition of skeletal muscle is broadly determined by regulatory systems that control the balance between muscle protein synthesis and degradation [1]. Skeletal muscle serves as the major protein and glycogen reservoir and the prime site for insulin-induced glucose disposal. In response to aging, inactivity, cancer or other advanced diseases, skeletal muscle mass is reduced, presumably to provide energy for physiological recovery. However, the net breakdown of muscle and the consequent loss of strength under such conditions can lead to metabolic dysfunction, and contribute to morbidity [2] and mortality [3].

Skeletal muscle is comprised of multiple fiber-type components that vary in their contractile properties, metabolic capacities and innervation by motor neurons. Based on myosin heavy chain (MyHC) isoform expression, adult mammalian myofibers can be subdivided into four major types: a slow, oxidative fiber expressing MyHC I and three fast glycolytic fibers expressing MyHC IIa, IIx, and IIb (in mouse). In humans, however, only type I, IIa and IIx myofibers are present [4]. There are also hybrid myofibers expressing mixed MyHC isoforms with intermediate characteristics (for example, MyHC IIa/IIx). Different myofiber types exhibit distinct transcriptional profiles. For example, transcripts enriched in the MyHC I fibers are primarily associated with ribosomal and contractile proteins, and enzymes involved in oxidative phosphorylation and fatty acid metabolism [5]. In contrast, genes expressed in MyHC IIb fibers tend to be more functionally diverse, but groups are associated with glycolysis/gluconeogenesis and insulin signaling [5]. Selective recruitment of different populations of fibers offers a wide range of performance capabilities from long lasting, low mechanical outputs with minimal fatigue (by slow, oxidative myofibers) to high-power, short-duration contractions (by fast, glycolytic myofibers). The fiber composition of a muscle is determined partly by genetic factors and is capable of changing in response to functional demands and stimuli. For example, resistance exercise induces functional and transcriptional changes targeted to the fast glycolytic fibers [6]. Moreover, myofiber type diversity affects the susceptibility of different muscles to muscle wasting state and disease. During aging, a preferential loss and atrophy of glycolytic, type II fiber is observed in both human and mice. Selective atrophy of type II [7] myofibers has also been reported in patients with steroid-induced myopathies [8], peripheral arterial disease [9], denervation [10], cancer cachexia [3], and respiratory failure [11]. Furthermore, several types of muscular dystrophy, such as Duchenne muscular dystrophy [12], and autoimmune diseases, such as myasthenia gravis [10], have been shown to induce muscle atrophy specifically in type II myofibers. Conversely, an association occurs between the loss of oxidative myofiber metabolism and insulin resistance [13]. However, recent studies in experimental systems have raised the possibility that the myofiber shift from oxidative to glycolytic metabolism may serve as an adaptive response to the diabetic state [14].

In contrast to the large number of molecular studies on oxidative fibers, there is a paucity of information on the roles that fast-twitch muscle fibers play in muscle diseases and metabolic function. To address this issue, we developed a conditional mouse model, dubbed the “MyoMouse”, that inducibly expresses a constitutively activated form of Akt1 specifically in skeletal muscle [15]. Akt1 is a serine/threonine kinase that regulates cell survival, growth, and metabolism in variety of tissues and cell types. In skeletal muscle, the Akt signaling pathway is activated during myogenic differentiation [16], by anabolic stimuli including growth factors (i.e., insulin and Igf1) [17], and by resistance exercise and nutritional inputs [18]. Induction of Akt1 signaling pathway leads to an increase in myofiber hypertrophy [17], whereas inactivation of Akt signaling leads to Foxo mediated muscle atrophy [19]. In the MyoMouse model, *Akt1* transgene activation promotes the selective hypertrophy of type IIb muscle fibers in a rapamycin-dependent manner in a subset of muscle groups [15]. Mice expressing the muscle-specific transgene display an increase in strength, but not an increase in running performance. Transgene activation in the MyoMouse model leads to a modest 5% increase in lean muscle mass, a physiologically relevant level that is on par with the muscle mass loss that occurs in the early stages of aging or disease [20].

A series of studies have used the MyoMouse model to examine the consequences of Akt1-mediated muscle growth in various models of chronic disease and acute injury. A relatively modest increase in myofiber growth in obese mice leads to marked reductions in fat mass and body weight, resolution of hepatic steatosis, and improvements in systemic metabolic parameters [15]. Notably, these metabolic improvements were associated with increased fatty acid oxidation in a remote tissue (i.e. liver), but not in muscle. Consistently, the restoration of muscle mass by *Akt1*-gene activation can correct age-associated impairments in systemic metabolism in mice fed a standard chow [20]. Other studies have shown that this model of muscle growth can have protective effects on distant organs in acute injury models of disease that are associated with the loss of skeletal muscle mass. Akt1-mediated muscle growth has been shown to protect against renal failure in two models of kidney injury [21], and diminish adverse cardiac remodeling following left anterior coronary artery ligation [22]. The MyoMouse model has also been employed to document the protective actions of acute *Akt1* transgene activation in muscle per se. Myogenic Akt signaling promotes sarcolemma stability and attenuates muscle degeneration in a model of Duchenne muscular dystrophy and improves regeneration in a cardiotoxin injury model [23, 24].

The striking changes observed in muscle and remote tissues of the MyoMouse have led us to speculate about the roles of “myokines”, i.e. hormonal factors released by muscle that confer some of the beneficial actions of exercise training [25, 26]. A number of strategies have been employed to isolate and characterize the muscle secretome involving, for example, comparisons of sedentary and exercised muscles [27], muscle growth following endurance training [28], muscle electrical stimulation [29], or development of lipid-induced insulin resistance [30], etc., and a number of myokine candidates have been identified. To date, a systematic analysis of the muscle secretome of the MyoMouse has not been performed, although this model exhibits a number of features that may provide unique insights. As discussed above, it is a model of selective fast-twitch fiber growth in mouse, and these are the myofibers that are preferentially lost in aging, sarcopenia and cachectic conditions. The effects of glycolytic muscle growth in this model are independent of exercise, nutritional input or surgical intervention, that can have confounding effects on the secretome. Finally, the effects of glycolytic muscle growth in the MyoMouse model is robust and rapid, potentially leading to an amplification in the levels of molecules involved in these regulatory events. Thus, to better characterize the cellular and molecular mechanisms involved in fast-twitch muscle growth, and its impact on the muscle secretome, we performed an in-depth and combined analysis of the transcriptome and metabolome on the growing muscles from the muscle-specific *Akt1* transgenic mice.

## Methods

### Animals

Skeletal muscle-specific conditional Akt1 transgenic mice (DTG) were generated by mating of 1256 [3Emut] Mck-rtTA [31] and Tre-myrAkt1 [32] transgenic mice as previously described [15]. All mice were genotyped by PCR from tail DNA. Mice were provided with chow and water ad libitum and housed in pairs on a fixed 12-h light/dark cycle in the Laboratory Animal Science Center at Boston University School of Medicine. At the age of 4 months, male DTG mice were treated with 0.5 mg/ml doxycycline (AB03550, American Bioanalytical) in drinking water for 2 weeks to induce skeletal muscle-specific Akt1 overexpression. To eliminate the effect of doxycycline water on muscle metabolism, Mck-rtTA or Tre-myrAkt1 single transgenic littermates, used as controls, were treated with doxycycline in the same manner as DTG mice. A day before, tissue harvest, body composition was assessed by non-invasive quantitative magnetic resonance (EchoMRI700, EchoMRI LLC, Houston, TX) at BUMC Metabolic Phenotyping Core. Mice were starved overnight before the day of sacrifice. Bilateral gastrocnemius muscles collected from anesthetized DTG and Mck-rtTA mice were weighed, snapped frozen in liquid nitrogen, and stored at−80 °C until analysis. All experiments were performed in adherence with NIH guidelines on the Use of Laboratory Animals, and were approved by the Institutional Animal Care and Use Committee at Boston University.

### RNA extraction and sequencing

Total RNA was isolated using TRIzol Reagent (Life Technologies, Grand Island, NY) according to manufacturer’s instructions followed by DNase I treatment using Qiagen (Valencia, CA) RNeasy Mini columns. The extracted RNA samples were analyzed using a BioAnalyzer and only high quality RNA samples (RIN > 8.5) were sent to Expression Analysis, Inc. (Durham, NC) for library preparation and sequencing (*n* = 4 per group). The mRNA library was prepared by Illumina TrueSeq stranded mRNA sample. Eight library preparations were loaded on two lanes of the Illumina HiSeq 2500 machines (Illumina, San Diego, CA) for paired-end 50 bp sequencing using the standard Illumina mRNA-seq protocol.

### RNA-seq data analyses

To prepare sequence reads for alignment, sequence adaptors were removed from sequences using Fastq-Mcf (code.google.com/p/ea-utils/wiki/FastqMcf). Several quality control steps were taken to assure quality, including the Illumina spike-in controls for each step of library prep, Life Technologies ERCC RNA spike-in control mix1 at the beginning of library prep, and UHRR control specimen for each plate batch of RNA-seq. Sequence reads with high-quality score (Phred score) of 33 and above were mapped to mm10 transcriptome using RNA-seq by Expectation Maximization (RSEM) v1.1.13 program [33]. After removal of the spike-in controls, an average of 59,760,883 reads were sequenced for each library preparation. There were ~88.1% sequence reads aligned to the transcriptome. At the gene level, aligned reads were annotated to 18,077 genes out of 30,743 genes defined by mm10 (58.8%). Genes with sequence reads less than 3 in at least 6 samples were considered as not detectable and thus filtered out from further analysis. All 8 samples were normalized by upper quartile normalization. Differential expression analysis was performed using edge-R where Fisher’s exact test was utilized. Genes or isoforms with false discovery rate (FDR) ≤ 0.005 and fold change ≥ 2 were considered as significantly differentially expressed genes (DEG) between two groups. The RNA sequencing data are available for download from the NCBI Gene Expression Omnibus database (GEO: GSE85763).

### Metabolomics

Unbiased metabolite profiling was performed using ion-pairing liquid chromatography/tandem mass spectrometry (Ion Pair LC/MS/MS) and gas chromatography tandem mass spectrometry (GC/MS/MS). Gastrocnemius was homogenized in methanol (100 mg/mL) by a ball-mill (MM301, Retsch GmbH, Haan, Germany), and centrifuged at 15,000 rpm for 5 min. 100 μL supernatant was mixed with sample buffer (5% octylamine, 3.5% acetic acid in 50% methanol), and centrifuged at 15,000 rpm for 5 min. The supernatant was transferred to a sample vial and placed in an autosampler (4 °C). For LC/MS analysis, each 10 μL sample was injected onto a reverse phase column from Atlantis T3 (2.1 × 100 mm, 3 μm, 130 Å, Waters co., Milford, MA, USA) and maintained at 35 °C. Subsequently, chromatographic separation was performed by gradient elution of mobile phase A, 0.1% octylamine, 0.07% acetic acid and 10 μM EDTA-2Na in MilliQ water, and mobile phase B, 0.07% acetic acid in methanol/isopropanol (4:1). The gradient started at 1% B for 2 min, increased up to 100% B in 10.5 min, maintained at 100%B for 4.5 min, then decreased to 1% B, and kept at 1% B for 10 min. Mass spectrometry analysis was performed using a QTRAP5500 mass spectrometer (AB Sciex Pte. Ltd., Toronto, Canada). The elution from liquid chromatography was directly introduced to electrospray ionization by using a Turbo spray ionization probe (AB Sciex Pte. Ltd., Toronto, Canada) with vaporizer temperature set at 475 °C. Multiple Reaction Monitoring (MRM) was used to detect the targeted molecules; where 148 molecules were detected by positive ionization and 188 molecules by negative ionization mode using simultaneous polarity switching. MRM conditions were set using reference conditions in the report by Yuan M et al. [34], with partially optimized standard reagents. LC/MS/MS data were processed by MultiQuant 3.0 (AB Sciex Pte. Ltd., Toronto, Canada), and the peak areas were exported to spread sheet and analyzed by Excel. For GC/MS/MS analysis, 50 μL of the homogenized supernatant were dried by nitrogen stream, and then derived by two-step reactions: oximation and trimethylsilylation. Oximation reaction was performed by adding 25 μL of O-methylhydroxylammonium chloride in pyrimidine (15 mg/mL), and incubated at 40 °C for 60 min. Subsequently, for trimethylsilylation reaction, 25 μL of N, O-bis (trimethylsilyl) trifluoroacetamide (BSTFA) with 1% trimethylchlorosilane (TMCS) was added to the reaction solution, and incubated at 60 °C for 60 min. The reaction mixture, 1 μL, was injected into an Agilent 7890A series gas chromatography system by split injection mode (10/1, v/v) using a GC injector 80 autosampler (Agilent Technologies Inc.). Gas chromatography separation was performed in a J&W Scientific DB-5MS-DG column (30 m × 0.25 mm i.d., df = 0.25 μm, Agilent Technologies Inc.) by temperature gradient, which rises at 10 °C/min from 60 °C to 325 °C, with consistent helium gas flow at 1 mL/min. The elution was ionized by electron impact ionization (70 eV) with an ion source temperature at 280 °C, and introduced to an Agilent 7010B triple-quadrupole mass spectrometer. Each target molecule was detected by MRM and the corresponding peak area was exported to an Excel spread sheet for further analysis. Differential expression analysis of the metabolites between the two groups were performed by Student’s *T*-test. Metabolites with *p*-value < 0.1 and fold change ≥ 1.5 or ≤ -1.5 were considered as statistically significant different.

### Pathway and functional analysis

Integrative pathway analysis for both differentially expressed genes and metabolites were performed using MetaboAnalyst 3.0 (www.metaboanalyst.ca) using default settings. Canonical signaling pathway and upstream regulator analysis were performed by uploading DEGs on to the Ingenuity Pathway Analysis package and analyzed using default settings (Qiagen, CA, USA).

### Secreted protein prediction and protein class annotation

To identify putative secreted proteins in Akt1-mediated muscle growth, five independent databases were queried. First, sequences of differentially expressed isoforms identified from RNA-seq analysis (*FDR* < 0.005 and fold change ≥ 1.5 or ≤ -1.5) were downloaded from UCSC Genome website (https://genome.ucsc.edu/cgi-bin/hgTables). Second, downloaded sequences were submitted to Signal P 3.1 Server (http://www.cbs.dtu.dk/services/SignalP/) to predict the presence of eukaryotic signal peptide at the N-terminus of protein using default settings. Third, sequences predicted to be absent of classical signal peptide were submitted to Secretome P 2.1 server (http://www.cbs.dtu.dk/services/SecretomeP/) to predict for the presence of non-classical signal peptide. Fourth, sequences positive for either classical or non-classical signal peptide were submitted to TMHMM server (http://www.cbs.dtu.dk/services/TMHMM/) to screen for transmembrane helices in protein sequence. Subsequently, sequences positive for either classical or non-classical secreted proteins but lack of transmembrane helices were submitted to WoLF PSORT database for a final screen for protein localization [35]. After removing sequences predicted to be predominantly located at places other than extracellular matrix (e.g. nucleus, mitochondria, endoplasmic reticulum, lysosome, and membrane, etc.) by WoLF PSORT, the remaining sequences are considered as a putative secreted protein, and thus a potential myokine. The list of DEG was uploaded to Panther Classification System for statistical overrepresentation test using PANTHER Protein Class annotation data set (http://www.pantherdb.org/). The heat map was generated by using GENE-E software from Broad Institute (Cambridge, MA).

### Immunohistochemistry

Medial and lateral gastrocnemius muscles were dissected, mounted separately on a wooden tongue depressor using OCT, immediately snap freeze in pre-chilled isopentane by liquid nitrogen, and stored at -80 °C. The frozen muscles were serially sectioned at 10 μM from the muscle mid-belly and mounted on microscope slides. For myofiber typing, unfixed sections were incubated at RT for 30 min, blocked by 10% BSA in PBST for 1 h, and incubated overnight at 4 °C with antibodies against MyHC isoform type I (BA.D5 mouse IgG2b), type IIA (SC.71 IgG1), type IIx (6H1 mouse IgG) from DSHB at 1:200 dilution. The next day, slides were washed and incubated with immunoglobulin-specific secondary antibodies at 1:200 dilution at room temperature (RT) for 1 h: Goat anti-mouse IgG2b AF350 (A21140), Goat anti-mouse IgG1 AF488, Goat anti-mouse IgM AF647 (A21238) from Invitrogen (Carlsbad, CA). Slides were post-fixed in ice-cold methanol for 10 min. After wash, sections were incubated with rabbit anti-mouse laminin antibody (L9393; Sigma) at 1:200 dilution in 1% BSA for 2 h at RT, followed by 1 h incubation in Texas-Red-X goat anti-rabbit IgG at 1:200 dilution (T-6491; Invitrogen) at RT. Sections were mounted on a coverslip with Vectashield mounting medium (H1000; Vector Lab, Burlingame, CA). For embryonic MyHC staining, sections were first fixed in 4% paraformaldehyde in PBS for 10 min and blocked with MOM Ig blocking reagent for 5 min (MKB-2213; Vector Lab). After washing, sections were incubated overnight in eMHC antibody from DSHB (F1.652 IgG2) at 1:1000 dilution, washed and incubated with Goat anti-mouse IgG2 AF488 at 1:200 dilution for 1 h. Sections were then stained for laminin as previously described and mounted in Vectashield mounting medium with DAPI (H1200) for imaging. For CD68 and F/480 staining, sections were first fixed and blocked as described above embryonic MyHC staining protocol. Sections were then incubated with Rat anti-mouse CD68 IgG2a or F4/80 IgG2b (at 1:100 dilution) with anti-laminin antibody (at 1:250 dilution) overnight at 4 °C. The next day, slides were washed, incubated with 1:250 dilutions of donkey anti-rat AF 594 and goat anti-rabbit IgG AF488 for 1 hour, and mounted in Vectashield mounting medium with DAPI for imaging. Images were captured with a BZ-9000 Keyence microscope camera at 20× magnification (Elmwood Park, NJ). Fiber cross-sectional area was circled manually and calculated by Keyence microscope. The average number of fibers measured for each myofiber type per muscle was 200.

### Statistical analyses

Statistical analyses of the RNA-seq and metabolomics data have been described above. All other statistical analysis was performed using GraphPad Prism 5.0 software (La Jolla, CA). For muscle weight, total RNA content and qPCR experiments, non-parametric Mann–Whitney test was used to determine whether there is a significant difference between the 2 groups. *P* < 0.05 is considered significantly different.

## Results

### Characterization of muscle growth induced by Akt1 transgene activation

**Fig. 1:**
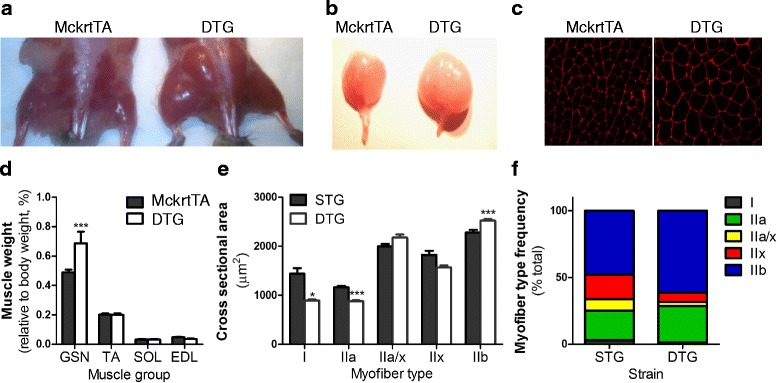
Two-weeks of *Akt1* transgene activation induces pronounced type IIb muscle growth in double transgenic (DTG) mice. **a** Skinned hind limbs of a 24-week-old MckrtTA and DTG mice after 2 weeks of doxycycline treatment to activate myr *Akt1* transgene expression. **b** A significant increase in the mass of gastrocnemius muscle was found in DTG mice at 2 weeks after transgene induction compared to MckrtTA mice. **c** Cross-section of the indicated gastrocnemius muscles stained with laminin (*red*). **d** Akt1-induction in DTG mice selectively increased the mass of gastrocnemius (GSN) muscle. **e** Average cross-sectional area of each myofiber type from DTG mice and their littermate controls (STG) with 2 weeks of doxycycline water. There were significant increases in CSA of type IIb fast-twitch myofibers but reduced CSA of type I and type IIa myofibers in DTG mice compared to STG controls. **f** Gastrocnemius muscles from DTG showed increased percentage of type IIb myofibers compared to that from STG mice. ***p < 0.001, *p < 0.05

To analyze the hypertrophy of skeletal muscle due to *Akt1* overexpression, gastrocnemius muscles were collected from mice that were positive for both Mck-rtTA and TRE-Akt1 transgene (i.e. double-transgenic (DTG) mice,) and Mck-rtTA single transgenic (MckrtTA) mice (also referred to as STG mice). Consistent with prior findings [15], 2 weeks of doxycycline treatment led to a significant induction in Akt1 phosphorylation in gastrocnemius muscles from DTG mice compared to control (Additional file 1: Figure S1A). This was associated with significant muscle growth and myofiber hypertrophy in DTG mice (Fig. 1a-c), and an 1.3-fold increase in gastrocnemius muscle weight (Fig. 1d). Because transgene activation only occurs in a small subset of muscle groups, there was no increase in muscle mass for tibialis anterior (TA), soleus (SOL) or extensor digitalis longus (EDL, Fig. 1d), that do not express the transgene, and no statistically significant change in body weight or body composition under these experimental conditions (Additional file 1: Figure S1B, C).

To better understand the consequences of acute Akt activation, an in-depth immunohistochemical staining analysis with various myosin heavy chain (MHC) antibodies was performed on gastrocnemius muscle sections. With regard to fiber cross-sectional area, there was a significant 9.5% increase in type IIb myofibers in the DTG versus control mice (Fig. 1e). Conversely, there were 38% and 24.3% reductions in type I and type IIa fibers, respectively. With regard to myofiber type frequency, *Akt1*-mediated gastrocnemius muscle growth led to a 27% increase in percentage of type IIb myofibers in DTG mice (Fig. 1f). These results confirm and extend our prior analysis of this model [15], and they define the degree of muscle growth following *Akt1*-transgene activation under the conditions employed for transcriptomic and metabolomic analyses employed in this study.

### Identification of differentially expressed genes (DEG) in hypertrophied muscle

**Fig. 2:**
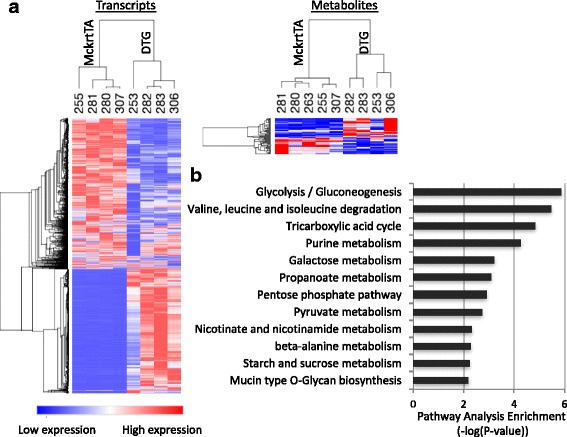
Data from poly (A+) RNA sequencing and metabolomics analyses of gastrocnemius muscle. **a** Heat map of transcriptional (*left panel*) and metabolite (*right panel*) changes of Akt1-mediated muscle growth. **b** Integrative pathway analysis of metabolomic and transcriptomic data for Akt1 induced muscle growth. Enriched metabolic pathways represented by both differentially expressed genes and metabolites are listed on the left

To understand the transcriptome changes associated with Akt1-mediated muscle growth, next-generation polyA (+) RNA sequencing was performed on the gastrocnemius muscle of DTG mice and their littermate MckrtTA control mice. We chose to study muscle growth at the 2-week time point because the muscle hypertrophy is at the beginning of log phase increase measured by muscle weight (Additional file 1: Figure S1d). Using statistical cutoff of FDR < 0.005 and fold change ≥2 or ≤−2, we identified 4,027 genes that were differentially expressed between *Akt1* transgenic muscle compared to control muscle. Among these, 2,009 genes were upregulated and 2,218 were downregulated (Additional file 2: Table S1). These DEG are displayed in the heat map in Fig. 2a (left panel) to illustrate the degree of reproducibility between mice.

### Metabolic pathways affected by the Akt1-mediated muscle growth

To understand the metabolome changes associated with Akt1-mediated muscle growth, untargeted metabolome was performed in gastrocnemius muscles using LC/MS/MS and GC/MS/MS platform. Among the 139 metabolites that could be detected, 29 were upregulated and 24 were downregulated using a statistical cutoff of *p*-value < 0.1, fold change ≥ 1.5 or ≤ -1.5 (Fig. 2a right panel, Additional file 3: Table S2). The combined transcriptomic and metabolomic data were analyzed using the MetaboAnalyst 3.0 data analysis tool [15] to identify the metabolic pathways that are enriched by Akt1-mediated muscle growth. Using this tool, metabolites and transcripts encoding for enzymes in the pathways associated with “glycolysis/gluconeogenesis”, “tricarboxylic acid (TCA) cycle”, “branched chain amino acid (BCAA) degradation”, and “pentose phosphate pathway”, among others, were significantly enriched in the muscle of DTG mice (Fig. 2b).

**Fig. 3:**
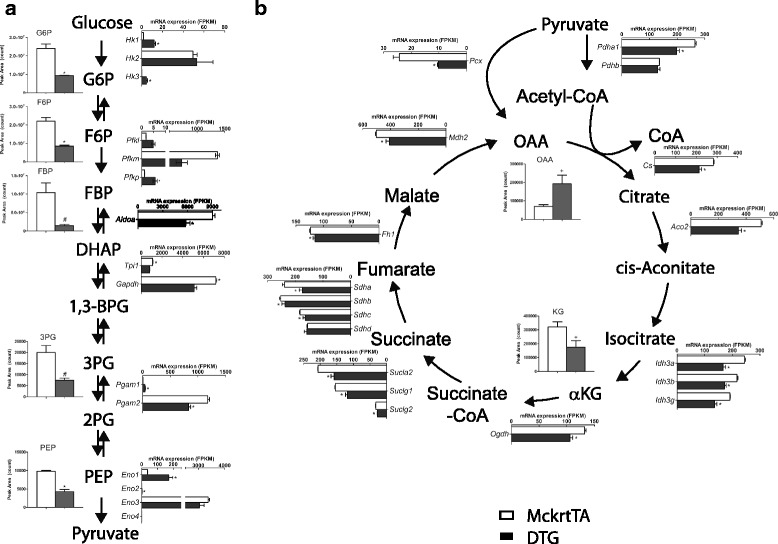
Schematic representations of the metabolite and transcript regulation in glycolysis and tricarboxylic acid cycle pathways. Regulated steps in glycolysis (**a**) and the TCA pathways (**b**) in response to Akt1-mediated muscle growth are shown. Metabolites with significant changes (from metabolome results) are shown as vertical bar graphs, whereas gene expression changes for enzymes (from RNA-seq results) are shown as horizontal bar graphs. Open column is control (MckrtTA) and filled column is DTG mice. * denotes *p*-value (for metabolites) or FDR (for transcripts) <0.005, # denotes *p*-value <0.05), and + denotes *p* < 0.1

Akt-mediated muscle growth was accompanied by reductions in the levels of many glycolytic pathway intermediates including glucose-6-phosphate (G6P), fructose-6-phosphate (F6P), fructose-1,6-bisphosphate (FBP), 3-phosphoglycerate (3PG), and phosphoenolpyruvate (PEP) (Fig. 3a). Correspondingly, transcripts encoding several enzymatic steps in the glycolysis pathway were downregulated, including aldolase A (*Aldoa*), triose phosphate isomerase 1 (*Tpi1*), glyceraldehyde-3-phosphate dehydrogenase (*Gapdh*), and phosphoglycerate mutase 2 (*Pgam2*) (Fig. 3a). In a number of cases, the downregulation of the transcript encoding the muscle isoform was accompanied by the corresponding upregulation of non-muscle isoforms. For example, a trend of downregulation of the muscle isoform *Pfkm* (FDR = 0.00539) was accompanied by the significant upregulation of the platelet (*Pfkp*) isoform. Similarly, the downregulated muscle-specific *Pgam2* was accompanied by the upregulation of *Pgam1*. Transcripts encoding the non-muscle isoforms were expressed at much lower levels relative to the muscle-specific transcripts, both in the control and transgenic muscle conditions.

Transcriptomic data indicated a strong decrease in mitochondrial tricarboxylic acid﻿ (TCA) cycle activity during Akt1-mediated muscle growth (Fig. 3b). Many transcripts encoding for enzymes in the TCA cycle were significantly downregulated including citrate synthase (*Cs*), aconitase 2 (*Aco2*), isocitrate dehydrogenase subunits (*Idh3a*, *Idh3b*, *Idh3g*), oxoglutarate dehydrogenase (*Ogdh*), succinate-coenzyme A ligase (*Sucla2*, *Suclg1*, and *Suclg2*), fumarate hydratase 1 (*Fh1*), *and* malate dehydrogenase 2 (*Mdh2*). Although few changes were detected in the TCA cycle metabolites, a 1.7-fold downregulation of α-ketoglutarate (α-KG) was observed.

Consistent with reductions in transcripts encoding TCA cycle enzymes, many genes encoding proteins required for oxidative phosphorylation were downregulated during Akt1-mediated muscle growth. Expression levels of 56 genes in the oxidative phosphorylation pathway, specifically those encoding for key enzymes in the electron transport chain, were downregulated more than 2-fold (Additional file 4: Figure S2 and Additional file 5: Table S3), including those encoding for alpha or beta subunits of NADH dehydrogenase (*Ndufa1*, *2*, *4*, *5*, *6*, etc., or *Ndufb2*, *4*, *6*, *7*, *8*, *11*), succinate dehydrogenase (*Sdha*, *Sdhb*, *Sdhc*), ubiquinol-cytochrome c reductase (*Uqcrc1*, *Uqcrc2*, *Uqcr10*, *Uqcr11*), cytochrome c oxidase (*Cox4l1*, *Cox4l2*, *Cox5a*, etc.), ATP synthase (*Atp5a1*, *Atp5b*, *Atp5c1*, etc.). Moreover, Akt1-mediated muscle growth also induced a significant downregulated expression in the transcripts encoding for mitochondrial fusion proteins (*Mfn1*, *Mfn2*, *Opa1*), mitochondrial permeability transition pore proteins (*Slc25a4*, *Vdac3*) and enzymes involved in reactive oxidative species detoxification (*Cat*, *Sod2*).

### Akt1-mediated effects on intermediate biosynthesis and degradation pathways

**Fig. 4:**
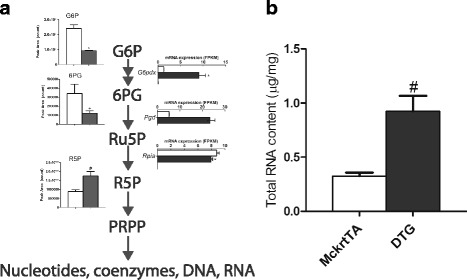
Regulation of pentose phosphate pathway metabolites and transcripts upon Akt1-mediated skeletal muscle growth. **a** Pathway regulation is indicated as described in the legend of Fig. 3. **b** Corresponding to an increase in pentose phosphate pathway flux, there was a significant 2-fold increase in total RNA yield per mg of muscle in Akt-mediated muscle growth (*p* < 0.05)

In contrast to these changes in the glycolytic and TCA pathways, there was an upregulation in the pentose phosphate pathway that functions in the production of intermediates for the biosynthesis of nucleotides and amino acids (Fig. 4a). Specifically, the transcript encoding glucose-6-phosphate dehydrogenase (*G6pdx*), the rate limiting enzyme in this pathway, increased 3.5-fold and phosphogluconate dehydrogenase (*Pgd*) increased 2.3-fold in the DTG muscle. Consistent with an increase in metabolite flux through the pentose phosphate pathway, a metabolite level crossover was detected by reductions in 6-phosphogluconic acid (6PG) and G6P and a 1.8-fold accumulation of ribose-5-phosphate (R5P). Increased flux through the pathway was also indicated by increases in purines and pyrimidine metabolites including AICAR and xanthosine (Additional file 6: Figure S3). Also consistent with the increase in the biosynthetic flux through this pathway, there was a marked increase in muscle tissue total RNA (Fig. 4b). Two weeks of *Akt1* transgene activation led to an ~2.8-fold increase in total RNA in gastrocnemius muscle (0.33 ± 0.03 μg/mg in MckrtTA vs. 0.92 ± 0.14 in DTG, *p* = 0.0286). The increase in tissue RNA was probably reflective of an increase in rRNA to meet the demand for increased protein synthesis.

**Fig. 5:**
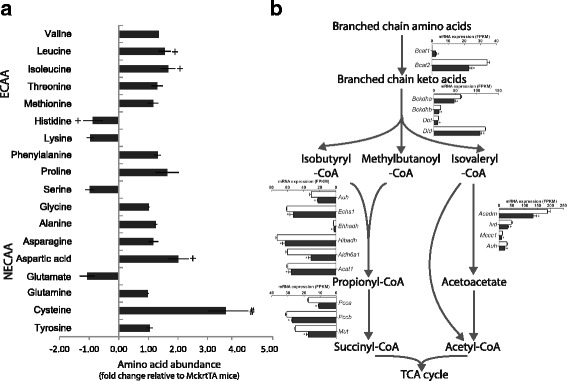
Changes in amino acid metabolism associated with Akt1-mediated muscle growth. **a** Relative levels of essential and non-essential amino acids (ECAA, NECAA) in the gastrocnemius muscles from DTG mice compared to those from MckrtTA mice. # denotes *p*-value <0.05, + denotes *p*-value <0.1. **b** Schematic representation of changes in the expression of transcripts encoding for branch chain amino acid degradation enzymes represented by histograms. Open column is control (MckrtTA) and filled column is DTG mice. * denotes transcripts with FDR <0.005

BCAA pathways were also enriched in the growing muscle. Accumulations of Ile and Leu (1.67- and 1.55-fold, respectively) were observed during Akt1-mediated muscle growth (Fig. 5a). Correspondingly, 17 genes encoding for enzymatic activities involved in the catalytic disposal of BCAA were downregulated (Fig. 5b), including the first 2 enzymes common to the degradation of three amino acids, branched-chain aminotransferase (encoded by *Bcat2*) and the rate limiting branched-chain α-keto acid dehydrogenase complex (encoded by *Bckdha* and *Bckdhb*). The primary BCAA aminotransferase isoenzyme responsible for initiating BCAA catabolism, mitochondrial Bcat2, that is predominantly expressed by muscle, was downregulated 2.9-fold at mRNA level. In contrast, the much less abundant cytosolic BCAT isoenzyme (*Bcat1*), which has roles other than BCAA oxidation, increased by 16.6-fold. In addition to the accumulation of BCAA, cysteine level increased 3.79-fold during Akt1-mediated muscle growth (*p* < 0.05), and increased Asp level was also observed (Fig. 5a).

### Signaling pathways involved in Akt1 mediated muscle growth

To identify the canonical signaling pathways mediating muscle growth, we performed Ingenuity Pathway Analysis (IPA) enrichment tests for the differentially expressed genes (Additional file 7: Table S4). Manual curating these enriched pathways suggested that “growth/cell cycle regulation” and “cellular immune response” are the major transcriptional signature in Akt1-mediated muscle growth (Table 1).

**Table 1:** Ingenuity analysis of canonical signaling pathways enriched by Akt1-mediated muscle growth

	Pathways	P	Ratio	z-score
Growth/Cell Cycle Regulation	Cyclins and Cell Cycle Regulation	2.38	0.31	3.64
	Integrin Signaling	4.83	0.30	2.78
	ILK Signaling	4.93	0.31	2.50
	Estrogen-mediated S-phase Entry	3.08	0.50	2.31
	Actin Cytoskeleton Signaling	4.22	0.28	2.06
	Mitotic Roles of Polo-like Kinase	3.02	0.35	2.00
	p70S6K Signaling	2.39	0.28	1.77
Cellular Immune Response	Fcγ Receptor-mediated Phagocytosis in Macrophages and Monocytes	9.02	0.46	4.42
	Dendritic Cell Maturation	5.19	0.32	4.24
	Leukocyte Extravasation Signaling	5.50	0.31	3.78
	Toll-like Receptor Signaling	4.19	0.38	3.77
	Role of Pattern Recognition Receptors in Bacteria & Viruses Recognition	4.02	0.32	3.40
	Production of Nitric Oxide & Reactive Oxygen Species in Macrophages	5.52	0.32	2.94
	TREM1 Signaling	6.64	0.45	2.65
	IL-8 Signaling	4.23	0.29	2.50
	NF-κB Signaling	4.15	0.30	2.38
	Tumoricidal Function of Hepatic Natural Killer Cells	2.51	0.46	2.12

P is reported as (-log (B-H *p*-value)). Ratio denotes the ratio of DEG specific to the pathway in this study divided by the total number of the genes in this pathway designated by IPA knowledge database. Z-score indicates a pathway with genes exhibition overall increased mRNA levels (positive value) or decreased mRNA levels (negative value)

**Fig. 6:**
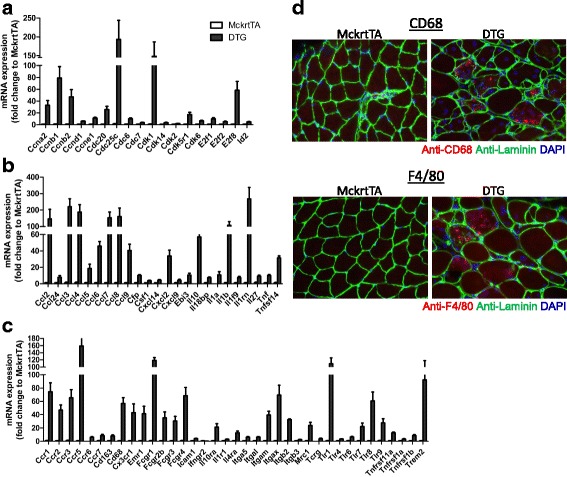
Increased expression of cell cycle regulators and pro-inflammatory transcripts during Akt1-mediated muscle growth. Transcripts with significant expression changes (fold change ≥ 2-fold, FDR < 0.005 for all transcripts) from RNA-seq results were represented in the histograms. **a** Increased expression of transcripts encoding for cell cycle regulators in Akt1-mediated muscle growth. **b** Expression changes of transcripts encoding for chemokines or cytokines in the gastrocnemius muscles of MckrtTA and DTG mice (fold change ≥ 2-fold, FDR < 0.005 for all transcripts listed). **c** Increased expression of the transcripts encoding for receptors of chemokines/cytokines and inflammatory cell markers (fold change ≥ 2-fold, FDR < 0.005 for all transcripts listed). **d** Immunohistochemical analysis showing the increased presence of inflammatory cell markers CD68 and F4/80 in gastrocnemius muscle from DTG mice compared to control STG mice

In the growth/cell cycle regulation category, “cyclins and cell cycle regulation”, “estrogen-mediated S-phase entry” and “mitotic roles of polo-like kinases” were predicted to be significantly activated pathways in the muscle of DTG mice (*p* < 0.001, Z-score 3.6, 2.3 and 2, respectively). Many transcripts encoding for cyclins, and cyclin dependent kinases were upregulated upon Akt1-mediated muscle growth (Fig. 6a). Consistent with the finding that cell cycle regulation pathways are enriched in the Akt1-mediated muscle growth, the IPA upstream regulator analysis algorithm also identified cyclin D1 as a nodal point regulator mediating the gene expression changes found in Akt1-mediated muscle growth (Additional file 8: Figure S4A). In this analysis, expression of 55 transcripts in Akt1-meidated muscle growth were directly regulated by cyclin D1 as predicted by the IPA literature knowledge base (*p* < 0.001, z-score 4.1, Additional file 8: Figure S4A), including several transcription factors important for cell cycle progression and proliferation (e.g. *Myc*, *Foxm1*, and *Uhrf1*). Notably, we also observed significant increase in the expression of *Myod1* (3.7-fold) and *Myog* (17.6-fold) transcripts that encode the transcriptional regulators of early muscle differentiation in the *Akt1* over-expressing muscle (Additional file 8: Figure S4B). In addition, our model of selective Akt1 induction in muscle is accompanied by an activation of embryonic program, indicated by the increases in transcripts encoding for isoforms associated with embryonic muscle development including cardiac troponin T type 2 (*Tnnt2*) or cardiac myosin binding protein c (*Mybpc3*), embryonic (*Myh3*) and neonatal myosin heavy chain (*Myh8*) (Additional file 8: Figure S4B). Immunohistochemical analysis confirmed that expression of embryonic myosin heavy chain (eMHC), encoded by *Myh3*, is readily detectable in the transgenic muscle following 2 weeks of Akt1 overexpression (Additional file 8: Figure S4C). Many of these eMHC-positive myofibers contained centralized nuclei, suggestive of satellite cell recruitment and the activation of a regenerative transcriptional program.

### Akt1-mediated muscle growth activates inflammatory pathways

Many of the differentially expressed genes are associated with Akt1-mediated muscle growth are modulators of inflammation (Table 1). Six of these enriched inflammation pathways listed in Table 1 are composed of genes that are expressed at very low levels, or below the level of detection, in the control muscle, suggesting that their marked upregulation during Akt1-mediated hypertrophy may be due to the infiltration of inflammatory cells. Several of these activated pathways share common DEGs encoding for pro-inflammatory cytokines including *Il1a*, *Il1b*, *Il10*, *Ccl2* and *Tnf* (Fig. 6b). Other highly upregulated transcripts were interleukin/chemokine receptors (e.g. *Il1r1*, *Il4ra*, *Ccr1*-*7*), members of toll-like receptors (e.g. *Tlr1*, *4*, *6*, *7*, *8*, *9*) and TNF-receptor family members (e.g. *Tnfrsf11a*, *1a*, *1b*) (Fig. 6c). The marked upregulation of inflammatory cell markers, such as *Cd68* and *Itgb2*, was also observed (Fig. 6c).

As the transcriptomic data suggested that monocytic cells infiltrate the rapidly growing muscle of DTG mice, immunohistochemical analysis of the gastrocnemius muscle sections was performed to assess for the presence of macrophages. The transgenic muscle mice showed positive staining for the macrophage markers CD68 and F4/80 (Fig. 6d). Little or no staining was detected in control muscle. The CD68+ and F4/80+ cells in the DTG mice tended to be localized to enlarged, irregular-shaped myofibers, many of which contained centralized nuclei. These results indicate that there is an inflammatory program in the transgenic mice that may contribute to the activation of regenerative myogenesis during Akt1- mediated muscle growth.

### Secreted protein transcripts induced by Akt1-mediated muscle growth

**Fig. 7:**
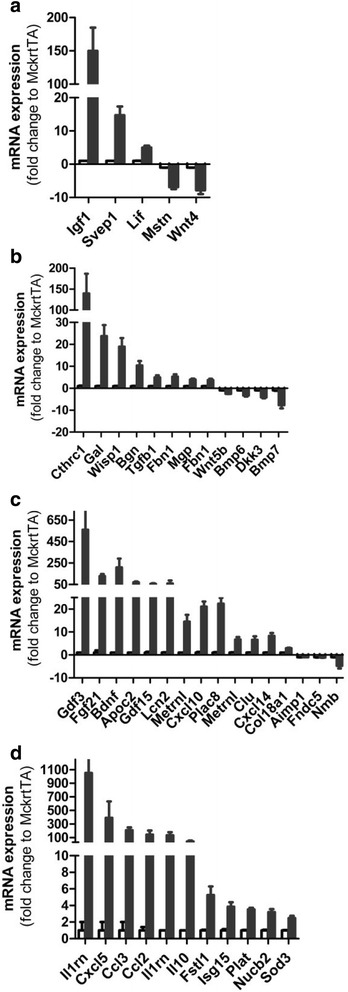
Akt1-mediated muscle growth alters the expression of transcripts encoding putative secreted proteins. RNA-seq profiling showed changes in factors implicated in the regulation of muscle growth (**a**), bone homeostasis (**b**), whole-body metabolism (**c**) and cardiovascular function (**d**) are shown. Open columns indicate control (MckrtTA) mice and filled columns indicate DTG mice. For all the transcripts listed, expression levels were changed with a fold change ≥ 1.5-fold or ≤ -1.5-fold and FDR < 0.005

It is well established that Akt overexpression in muscle leads to changes both in the muscle and at remote tissues [15, 20–24]. To explore the hypothesis that these changes may be coordinated by secreted proteins, candidate muscle-derived secreted proteins were analyzed in the transcriptome data set. At the 2 week time point, 174 differentially regulated transcripts encoding putative secreted proteins were upregulated, whereas 66 were downregulated (Additional file 9: Table S5). A Panther over-representation test was performed to determine the protein classes represented by these differentially expressed secreted proteins. This analysis revealed that most of these encoded proteins could be classified as chemokines, cytokines, extracellular matrix proteins, protease or protease inhibitors (Additional file 10: Table S6). Among these putative secreted proteins, several of the Akt1-mediated secreted proteins have been previously characterized to mediate muscle growth/myogenesis (Fig. 7a), bone homeostasis (Fig. 7b), metabolism (Fig. 7c), and cardiovascular functions (Fig. 7d). Some of the proteins encoded by these transcripts have been shown to be secreted into the circulation during exercise or muscle growth, such as *Igf1* [36], *Fstl1* [37], *Metrnl* [38], and *Bdnf* [39].

## Discussion

A small but growing series of studies have shown that increases in fast-twitch skeletal muscle mass, with no increase in oxidative capacity, can lead to improvements in systemic metabolism [18]. Furthermore, it is becoming increasingly appreciated from clinical studies that resistance exercise, that leads selectively to the growth of faster-twitch muscle, can diminish the risk of cardiovascular disease [2, 40]. However, the cellular and molecular mechanisms by which fast-twitch muscle exerts these effects are poorly understood. Some of the beneficial effects of resistance exercise on whole-body metabolism can be attributed to the increase in calorie consumption for maintaining muscle mass. In addition, the growing muscle can secrete peptides or metabolites into circulation that may affect remote metabolic and cardiovascular tissues. Previously, our lab has reported that a murine model of fast-twitch glycolytic muscle growth that is triggered by the inducible expression of a constitutively active *Akt1 *isoform. These mice display accelerated muscle regeneration [23, 24], resistance to systemic metabolic dysfunction [15] and preserved function in models of cardiometabolic and renal diseases [21, 22]. Thus, to better understand the cardiometabolic-protective properties of Akt1-mediated muscle growth we performed RNA sequencing and non-targeted metabolomic analyses on this muscle tissue from this model.

Consistent with our previous report [15], there was a 30% increase in the mass of fast/glycolytic (gastrocnemius) muscle from transgenic mice with 2 weeks of Akt1 overexpression, that was accompanied by an increase in the mean cross sectional area of type IIb myofibers and an increase in the distribution of IIb myofibers. Akt1 is a multifunctional kinase that integrates signals from nutrients, growth factors, energy status and environment stresses to control cellular growth. Prior studies have shown that Akt1 is a regulator of muscle growth through activation of mTOR signaling [17, 41]. In the current study, the P70S6K signaling pathway, a canonical downstream substrate of mTOR, was found to be enriched (Table 1), and it is likely to contribute to Akt1-mediated muscle growth.

Combining transcriptional/metabolomic data, we identified the robust upregulation of biosynthetic metabolic pathways and the downregulation of catabolic pathways in Akt1-mediated muscle growth. Intermediates that comprise the core metabolic pathways of glycolysis and the TCA cycle were depleted in the growing muscle of the Akt1-mediated muscle growth, consistent with a large demand for intermediates required for the synthesis of proteins and nucleic acids. The pentose phosphate pathway was one of the most significantly enriched metabolic pathways in the growing muscle. Indeed, changes in purine metabolism and increased total RNA content in the MyoMouse muscle, suggests that enhanced flux through the pentose phosphate pathway is integral in reprograming the tissue to support rapid type IIb muscle growth. There was a significant 2-fold increase in the level of the R5P intermediate and increased mRNA expression of the first 2 enzymes in the pathway, *G6pdx* and *Pgd. G6pdx* encodes for the rate-limiting enzyme of the pathway, catalyzing the first irreversible reaction that leads to the generation of R5P, a precursor for de novo nucleotide biosynthesis [42]. The finding of increased flux through the pentose phosphate pathway upon Akt1-mediated muscle growth is consistent with previous findings showing that *G6pdx* transcriptional activation is dependent on insulin/PI3K/Akt/mTOR signaling [43]. It has also been reported that enzyme activities of *G6pdx*- and *Pgd*-encoded proteins are significantly induced during muscle regeneration and that this induction can be reversed by administration of cycloheximide and actinomycin D [44]. Collectively, these results suggest that Akt1-mediated muscle growth is dependent on enhanced pentose phosphate pathway flux to support the rapid muscle growth.

Akt1-mediated muscle growth led to increased leucine and isoleucine accumulation, and this was accompanied by the diminished expression by transcripts that encode for BCAA degradation enzymes. BCAAs, and in particular leucine, are strong activators for muscle protein synthesis [45, 46]. Reductions in transcripts encoding BCAA catabolic enzymes have been reported in other studies, such as muscle growth in response to synergic ablation [47]. Notably, the significant transcriptional suppression of BCAA degradation enzymes in Akt-mediated muscle growth was in marked contrast to the muscle-specific *PGC1α *transgenic mice that display a uniform increase in the transcripts that encode BCAA catabolic enzymes [48]. It was proposed that upregulation of BCAA catabolic enzymes (i.e. *Hibch* and *Hibadh*) in the muscle-specific *PGC1α *transgenic mice results in muscle secretion of a BCAA catabolic intermediate, 3-hydroxyisobutyrate, which activates endothelial fatty acid transport and promotes lipid accumulation in muscle, leading to insulin insensitivity in this model [48]. In contrast, the *Akt1* transgenic mice showed a 2.2-fold decrease in *Hibadh* mRNA and an overall downregulated BCAA catabolism, which provide a mechanistic explanation for the differences in insulin sensitivity observed in these two models of high-fat diet fed transgenic mice [15].

Cyclins and cell cycle regulation are other functional categories enriched in the DEG analysis of Akt1-mediated muscle growth. Using the Upstream Regulator Analysis in the IPA software package, Cyclin D1 was identified as a significant regulator mediating transcriptional changes in 55 genes during Akt1-mediated muscle growth (Additional file 8: Figure S4A). These results are consistent with the satellite cell activation accompanied by the improvement in muscle regeneration previously described with this model [23]. The finding of cell cycle activation in the Akt1-mediated muscle growth is also consistent with a recent human cohort study, where 16-weeks of resistance training induced upregulation in Cyclin D1 expression, and higher levels of satellite cell activation in the individuals with robust muscle hypertrophy, but not in the non-responders [49]. Increased myonuclear number and satellite cell content occurs during muscle hypertrophy in humans, particularly in individuals with high extents of muscle growth [50, 51]. Consistently, Akt1-mediated mTOR signaling has been shown to be important for priming satellite cells for cell cycle entry [52] and for the expression of myogenic factor such as myogenin and MyoD (Additional file 8: Figure S4B) [53]. In the current study, muscle growth was associate with a 3.7-fold increase in *Myod1* mRNA and a 17-fold increase in *myog* mRNA. MyoD is known to epigenetically regulate muscle cytoskeletal proteins expression [54] and promote expansion of muscle progenitor cells. These findings are consistent with a report showing that Akt1 increases myonuclei number per fiber by promoting MyoD-mediated myogenic transcription regulation during myoblast differentiation [55]. Finally, immune-histochemical analysis revealed a significant increase in embryonic-like muscle fibers during in Akt1-mediated muscle growth (Additional file 8: Figure S4C) accompanied by a trend of increase in embryonic and neonatal myosin heavy chain (*Myh3* and *Myh8*). Thus, we speculate the increased expression of embryonic and neonatal myosin heavy chain in the transgenic muscle is a result of robust muscle progenitor cell proliferation, followed by cell cycle arrest and differentiation.

Emerging evidence indicates that induction of fetal sarcomeric proteins in adult tissues is triggered by or associated with the altered expression of the key metabolic enzymes [56, 57]. In adult failing heart, for example, the expression of fetal sarcomeric proteins is accompanied by metabolic switching to more closely resemble the fetal heart, such as a switch from fatty acid metabolism to anaerobic glycolysis, which is thought to protect against functional impairment and cell death [57]. While it is well-established that skeletal muscle alters myofiber type in response to stress, the consequences of metabolic shifts during fetal re-programing is less known. In activated satellite cells, genes regulating glycolysis are upregulated [58, 59], and it has also been shown that byproducts of glycolysis are required for successful differentiation of C2C12 murine myoblasts [60]. Our observations of reduced glycolysis in Akt1-mediated muscle growth implies impaired myogenesis. However, since satellite cells account for only 3–5% of the total number of myofiber nuclei, their contribution to the observed changes in overall metabolite levels should be small. On the other hand, the activation of other cell types besides satellite cells, such as myofibroblasts, fibroblasts, pericytes, or endothelial cells, may contribute to the enrichment of pathways associated with cell cycle progression and the metabolic shift in the *Akt1*-transgenic muscle [56, 61]. In this regard, Akt-mediated muscle growth was associated with an activation of non-muscle isoforms of metabolic enzymes whereas the muscle isoforms were downregulated in the tissue. This switch from muscle to non-muscle isoform could reflect a metabolic adaptation associated with the de-differentiation of the muscle tissue. Alternatively, it could reflect greater recruitment of non-muscle cell types to the tissue, such as inflammatory cells, that predominantly express the non-muscle isoforms.

Akt1-mediate muscle growth led to a strong signature from inflammatory modulators in the DEG analysis. Notably, some of the enriched inflammatory pathways are known to be functional in skeletal muscle. For example, enriched DEGs occurred in the “production of nitric oxide and reactive oxygen species in macrophages” pathway. This category included both cytosolic and membrane bound subunits of NADPH oxidase in the Akt1- mediated muscle growth (*Nox4*, *gp*-*91 phox*, *p22 phox*, *p47 phox* and *p67 phox*). Increased membrane translocation of NADPH oxidase has been shown to be one of the key sources of reactive oxygen production in contracting muscle [62]. It has been proposed that growth factor-mediated transient increases in H_2_O_2_ can improve insulin sensitivity by inhibiting the action of protein tyrosine phosphatases [63, 64], consistent with the phenotype of the *Akt1* transgenic mouse. Inflammation responses have also been associated with muscle hypertrophy in a number of studies, and it is thought to influence muscle regeneration by directly affecting satellite cells and indirectly through the modulation of angiogenesis and fibrosis [65, 66].

There is mounting evidence that skeletal muscle is an endocrine tissue capable of releasing bioactive peptides or proteins that have been referred to as “myokines” [25, 26]. In the current analysis we identified 240 differentially-regulated transcripts encoding a putative signal peptide and lacking of a transmembrane spanning domain. A number of the differentially regulated transcripts encode for proteins that have previously been shown to regulate muscle growth, satellite activation, or myogenesis (Fig. 7a). For example, the expression of insulin-like growth factor 1 transcript variant 2, (*Igf1*, uc007gqx.2, containing Class II signal peptide and terminating at exon5) is increased by 176-fold during the Akt1-mediated muscle growth. Igf1 is known to act both on muscle fibers and on activated satellite cells to induce hypertrophy [67]. Notably, clinical trials have demonstrated that recombinant human IGF-I administration can improve insulin sensitivity in type I or type II diabetes [68, 69]. We also found that leukemia inhibitory factor (*Lif*) transcripts are upregulated during Akt1-mediated muscle growth. Previous studies have shown *Lif* mRNA expression is modestly increased 6-h after resistance exercise in human muscle biopsy specimens and secreted into medium 3-h after electrical stimuli of myotubes [70]. Lif has been shown to promote myogenic cell proliferation [70], muscle glucose uptake [71], and is required for overload-induced muscle hypertrophy [72]. Myostatin (*Mstn*), a widely studied negative regulator of muscle growth [73], was downregulated 7.3-fold at the transcription level in Akt1-mediated muscle growth. These findings are consistent with a study showing that *Mstn* mRNA is downregulated by resistance exercise in human muscle [74].

It was of interest to assess whether the *Akt1* transgenic model was capable of regulating transcripts in muscle that could potentially be involved in bone homeostasis. The analysis also identified several regulated transcripts encoding for proteins implicated in bone growth and protection (Fig. 7b). Collagen triple helix repeat containing 1 (*Cthrc1*) transcript increased 84-fold in Akt1-mediated muscle growth. *Cthrc1* encodes for a secreted protein that was first identified to increase bone mass by regulating osteoblast proliferation and differentiation [75]. Recent studies also show that this factor has a metabolic function, as whole-body *Cthrc1* deficiency will promote liver steatosis and increased subcutaneous fat mass [76, 77]. Another example is biglycan (encoded by *Bgn*) that is induced 11-fold by Akt1 in muscle. Biglycan is a member of small leucine-rich proteoglycan family that is abundantly expressed in mineralized tissues. Whole-body *Bgn*-deficient mice display defective bone formation and mineralization [78], and biglycan has been shown to promote proper blood vessel formation during fracture repair [79] and regulate muscle-tendon formation during development [80]. It has also been shown that biglycan impedes progression of atherosclerosis by mitigating thrombin activities and inflammation, suggesting that this factor can have other systemic actions [81].

Akt1-mediated muscle growth leads to the increased expression of several transcripts encoding metabolic-regulatory proteins (Fig 7c). Among these, the transcript encoding *Fgf21* was robustly upregulated (250-fold), consistent with a previous report [82]. Fgf21 functions as a metabolic regulator capable of lowering blood glucose in animals with diabetes, although this effect was not observed in clinical trials [83]. It has also been shown that Fgf21 induction decreases plasma triglycerides in rodent and human [84, 85], and that it has cardio-protective actions in mice [86]. It has been reported that serum FGF21 level increased 1 hour after a moderate to high intense treadmill exercise [87], but several reports suggested that liver instead of the skeletal muscle is the main endogenous source after exercise. In addition, Meteorin-like protein (*Metrnl*) was upregulated during Akt1-induced muscle growth. Meteorin-like protein was shown to be induced in muscle by the overexpression of *PGC1*-α*4* [38]. This factor can be induced in human muscle after a bout of combined resistance and endurance exercise, and it is secreted into circulation in mice after an acute bout of downhill running exercise. In addition, several myokines identified from the transcriptome data in Akt1-mediated muscle growth have been previously shown to regulate energy homeostasis by controlling patterns of feeding and neural circuits. For example, *Bdnf*, a member of the neurotrophin family, increased 153-fold at the mRNA level in the Akt1-mediated muscle growth. Both human and mouse data showed that decreased brain-derived neurotrophic factor (BDNF) is associated with the hyperphagia, development of obesity and neurodegenerative disease [88, 89]. BDNF and its cognate receptor TrkB have been implicated to regulate food intake and appetite by targeting at ventromedial nuclei in the hypothalamus and dorsal vagal complex in the brainstem [89, 90]. Multiple tissues and cell types express BDNF [90]. In skeletal muscle, BDNF overexpression increases fatty acid oxidation in an AMPK-dependent manner and circulating BDNF is increased after exercise [39]. *Fndc5*, the transcript that encodes the putative irisin precursor protein, decreased 2-fold in transcript level in the Akt1-mediated muscle growth. Although controversial, modest increases in circulating irisin levels were observed after exercise training in both mouse and human [91], and it is reported to improve cognitive function by inducing BDNF and other neuroprotective genes in hippocampus [92].

Akt1-mediated muscle growth also led to the induction of several factors that are known to regulate cardiovascular function (Fig. 7d). Several of these have been shown to be protective in models of myocardial ischemia-reperfusion injury including Il1rn, Fstl1, Isg15, Plat, Ncub2 and Sod3. Notably, the transcript encoding for follistatin-like 1 (*Fstl1*) increased by 5.7-fold during Akt1-mediated muscle growth. FSTL1 is upregulated in skeletal muscle by strength-training in individuals [93], and increased circulating FSTL1 was found after 60 min of cycling exercise [37]. Numerous studies have shown that Fstl1 has cardiorenal-protective properties [94]. *Plat* mRNA was increased 3.5-fold in the transgenic muscle. *Plat* encodes tissue-type plasminogen activator (t-PA), which converts the zymogen plasminogen into proteolytically active serine protease plasminogen that degrades fibrin clots and has been shown to be an effective acute treatment in acute myocardial infarction [95]. Elevated circulating t-PA has been shown after both acute endurance and resistance exercises in human [96]. Although endothelium is the main site of t-PA production, t-PA is present at low levels in normal mouse muscles and it is induced during muscle regeneration [97]. This transcriptomic analysis of muscle-specific *Akt1* transgenic mice identified a number of putative myokines that are regulated during fast-twitch/glycolytic muscle growth. While several of these myokine candidates have been studied previously, the majority of these proteins have yet been characterized in the context of muscle growth or cardio-metabolic regulation.

## Conclusion

In summary, we have utilized an inducible, muscle-specific *Akt1*-transgenic mouse system to characterize metabolic and gene expression profiles associated with fast-twitch muscle growth. These results revealed that muscle growth was accompanied by reduced glycolysis and oxidative phosphorylation, and the activation of pathways that favor the biosynthesis of macromolecules. Previous work with this model of muscle growth have documented enhanced sarcolemma stability during muscle degeneration, improved whole-body metabolism in diet-induced obesity, and tissue protection in models of heart failure and kidney injury. Unbiased profiling identified numerous transcripts encoding secreted proteins that may confer some of the systemic protective actions of fast-twitch skeletal muscle.

## Additional files

Additional file 1: Figure S1. Doxycycline led to a significant induction in AKT1 and growth in gastrocnemius from DTG mice. (PDF 113 kb)
Additional file 2: Table S1. List of differentially expressed genes in gastrocnemius muscles from MckrtTA and DTG mice. (XLSX 726 kb)
Additional file 3: Table S2. List of differentially expressed metabolites in gastrocnemius muscle in MckrtTA and DTG mice. (XLSX 31 kb)
Additional file 4: Figure S2. A schematic representation of gene expression changes in the oxidative phosphorylation pathway. (PDF 590 kb)
Additional file 5: Table S3. List of down-regulated genes in oxidative phosphorylation pathway in DTG mice compared to MckrtTA mice. (XLSX 44 kb)
Additional file 6: Figure S3. Level of purine (A) or pyrimidine (B) metabolites in the gastrocnemius muscles. (PDF 35 kb)
Additional file 7: Table S4. Excel data source file for Table 1 with DEG names. (XLSX 36 kb)
Additional file 8: Figure S4. Cell cycle regulator-mediated signaling and embryonic myogenesis are activated in Akt1-mediated muscle growth. (PDF 483 kb)
Additional file 9: Table S5. Full list of secreted proteins altered by Akt1-mediated muscle growth. (XLSX 64823 kb)
Additional file 10: Table S6. GO analysis of the secreted proteins altered in Akt1-mediated muscle growth. (XLSX 46 kb)

## Abbreviations

Akt1: RAC-alpha serine/threonine-protein kinase (PKB alpha)
Angptl4: Angiopoietin-like factor 4
BCAA: Branched chain amino acid
Bdnf: Brain-derived neurotrophic factor
Ccnd1: Cyclin D1
Cthrc1: Collagen triple helix repeat containing 1
DEG: Differentially expressed genes
DTG: 1256 [3Emut] Mck-rtTA and TRE-myrAkt1 double transgenic mice
Fndc5: Fibronectin type III domain containing 5 (Irisin)
Fgf21: Fibroblast growth factor 21
Fstl1: Follistatin like 1
G6pdx: Glucose-6-phosphate dehydrogenase
GO: Gene ontology
GSN: Gastrocnemius muscle
Igf1: Insulin like growth factor1
Ile: Isoleucine
IPA: Ingenuity pathway analysis
Lcn2: Lipocalin 2
Leu: Leucine
Lif: Leukemia inhibitory factor
MckrtTA: 1256 [3Emut] Mck-rtTA single transgenic mice
Metrnl: Meteorin-like protein
mTOR: Mechanistic target of rapamycin
MyHC: Myosin heavy chain
Myod1: Myogenic differentiation 1 (MyoD)
Myog: Myogenin
NADPH: Nicotinamide adenine dinucleotide phosphate-oxidase
P70S6K: Ribosomal protein S6 kinase beta-1
PGC1α: Peroxisome proliferator-activated receptor gamma, co-activator 1 alpha
Pgd: Phosphogluconate dehydrogenase
PPP: Pentose phosphate pathway
R5P: Ribose 5 phosphate
RNA-seq: RNA sequencing
STG: 1256 [3Emut] Mck-rtTA or TRE-myrAkt1 single transgenic mice
Svep1: Sushi, von Willebrand factor type A, EGF and pentraxin domain containing 1
TCA: Tricarboxylic acid cycle
Val: Valine
