# Supplementary figures and images for: RNA-seq and metabolomic analyses of Akt1-mediated muscle growth reveals regulation of regenerative pathways and changes in the muscle secretome
Source: BMC Genomics. 2017 Feb 16;18:181. doi: 10.1186/s12864-017-3548-2 (PMC5314613; doi:10.1186/s12864-017-3548-2)

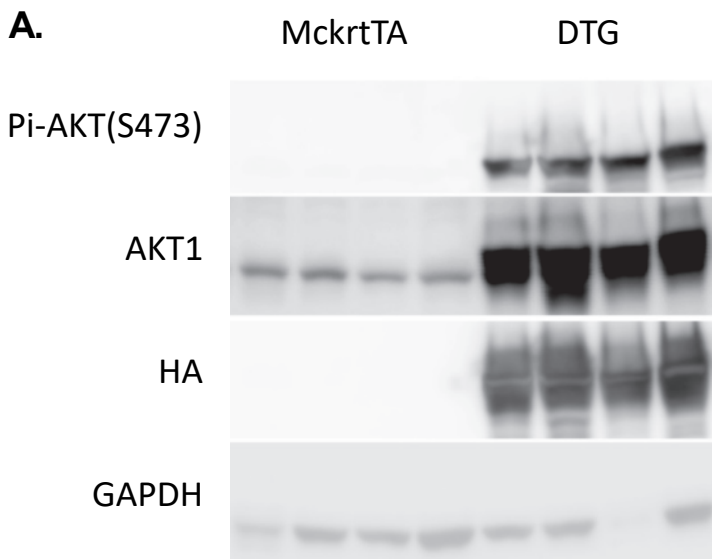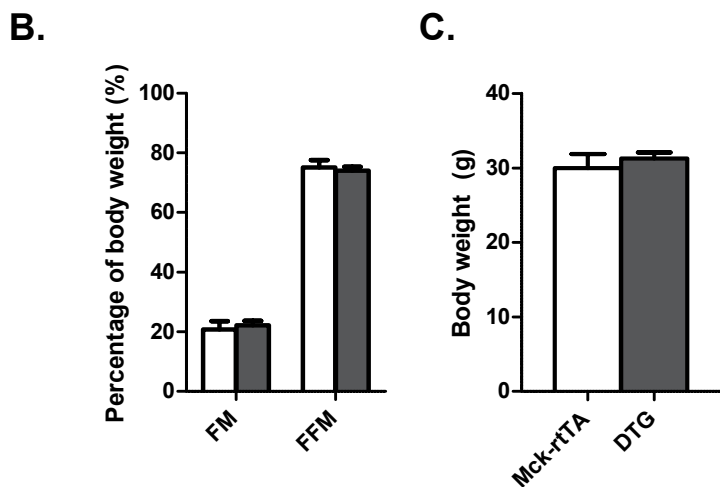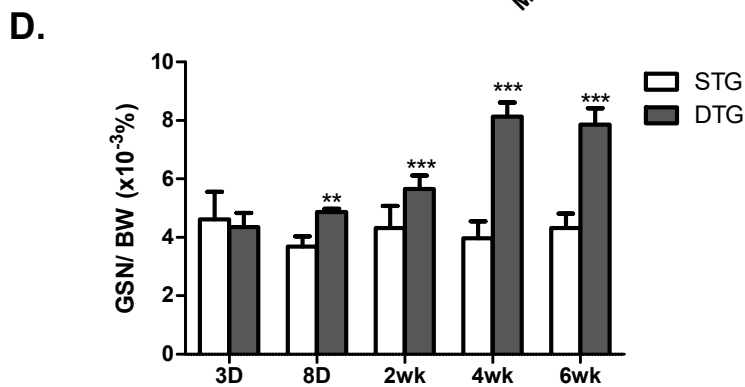

Supplement: Additional file 1: Figure S1. — Doxycycline led to a significant induction in AKT1 and growth in gastrocnemius from DTG mice. (PDF 113 kb) [file 12864_2017_3548_MOESM1_ESM.pdf]

## Additional File 4

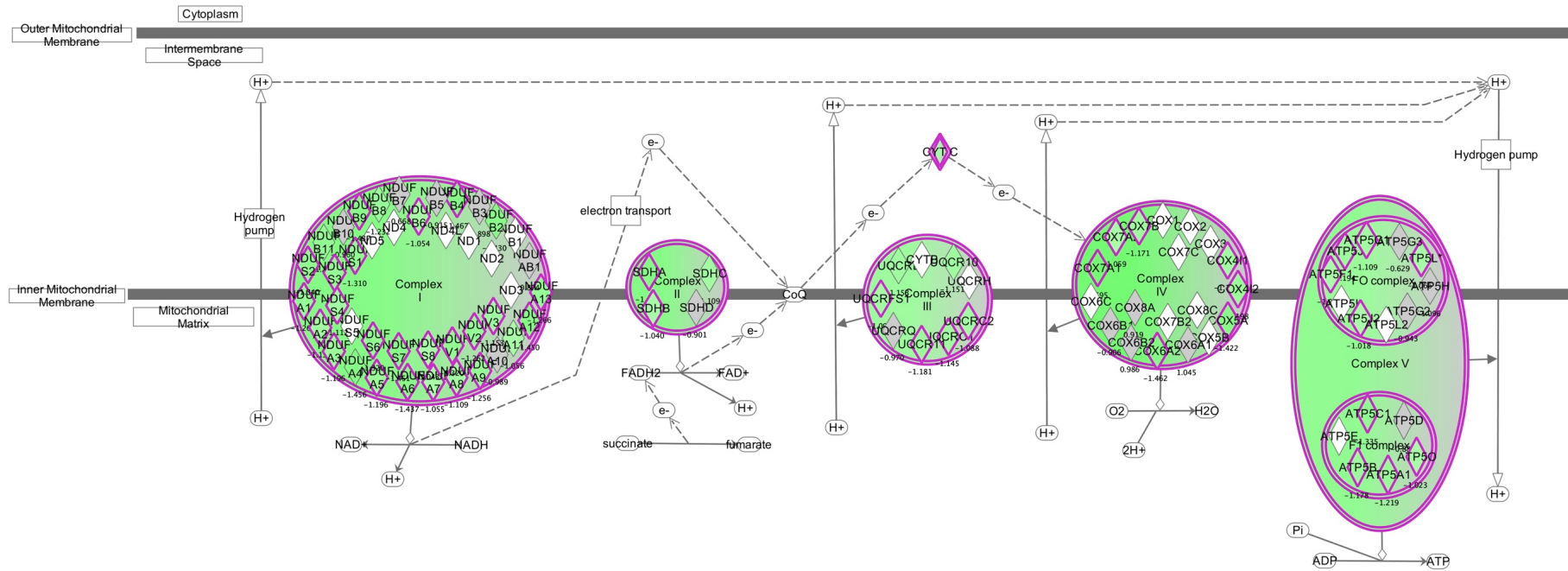

Supplement: Additional file 4: Figure S2. — A schematic representation of gene expression changes in the oxidative phosphorylation pathway. (PDF 590 kb) [file 12864_2017_3548_MOESM4_ESM.pdf]

**A.**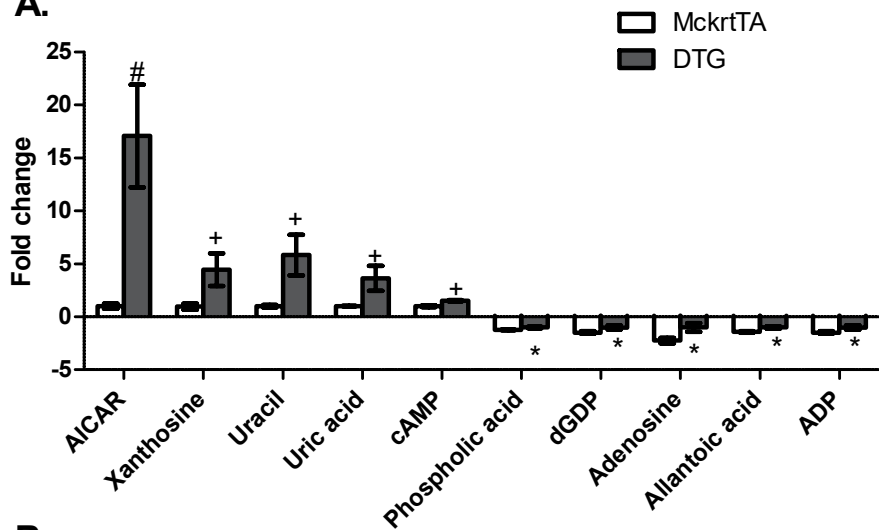**B.**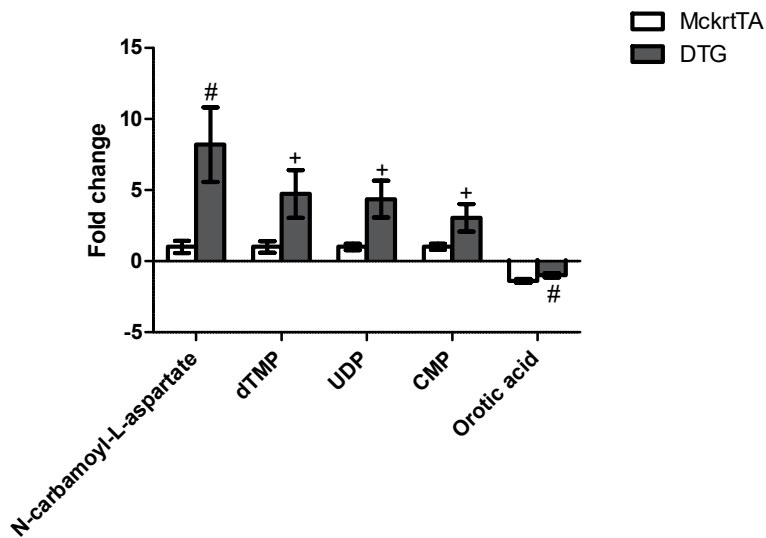

Supplement: Additional file 6: Figure S3. — Level of purine (A) or pyrimidine (B) metabolites in the gastrocnemius muscles. (PDF 35 kb) [file 12864_2017_3548_MOESM6_ESM.pdf]

# Additional File 8

A.

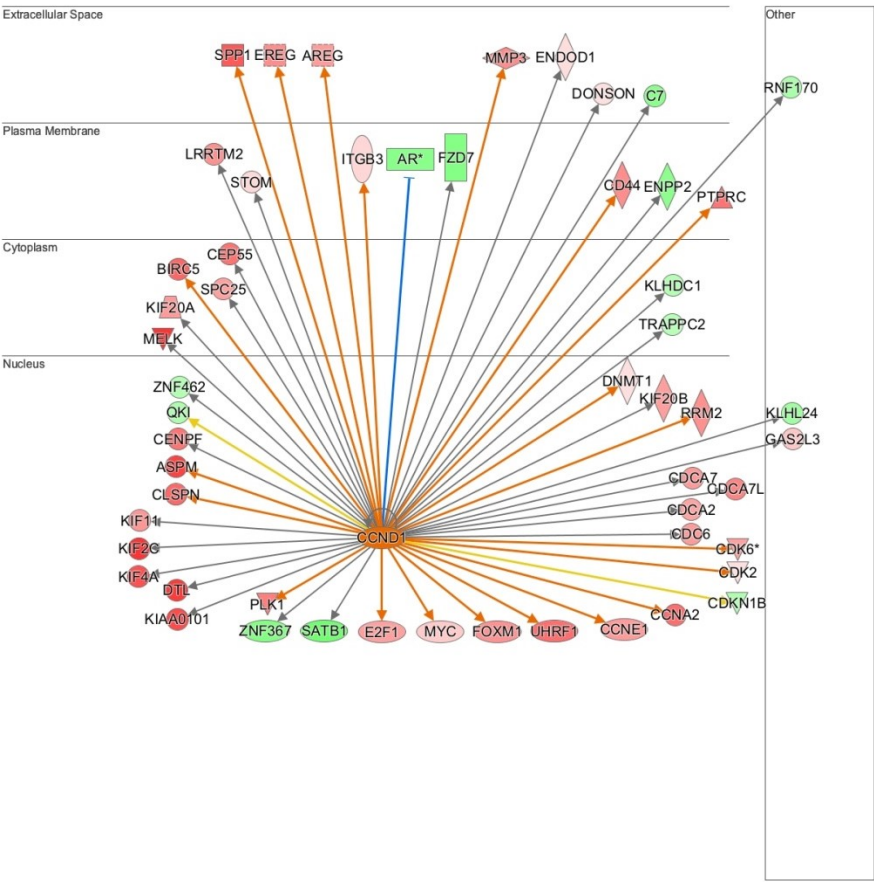

B.

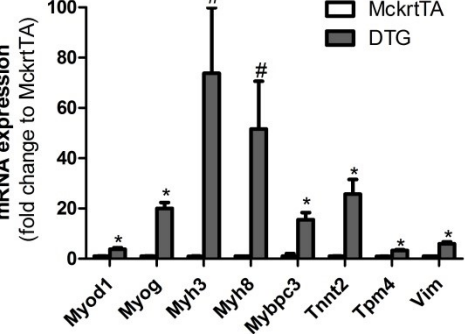

C.

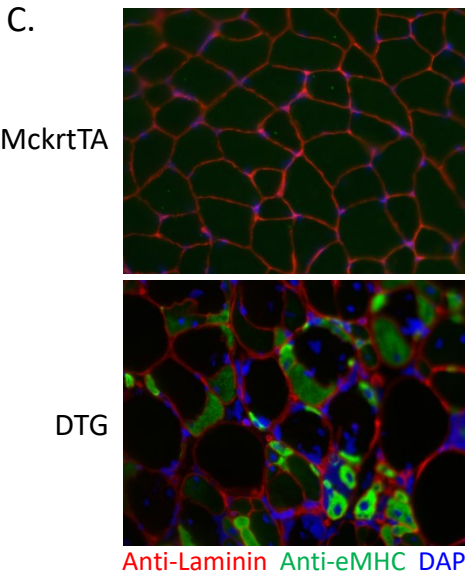

Supplement: Additional file 8: Figure S4. — Cell cycle regulator-mediated signaling and embryonic myogenesis are activated in Akt1-mediated muscle growth. (PDF 483 kb) [file 12864_2017_3548_MOESM8_ESM.pdf]
